# Investigating the causal role of cellular senescence-related genes in preeclampsia: a multi-omics Mendelian randomization study with differential expression analysis

**DOI:** 10.3389/fendo.2025.1661666

**Published:** 2025-10-27

**Authors:** Hao Zhu, Yi Yu, Jue Wang, Chengjie Wang, Zhenzhen Liu, Xiaoyue Zhang, Rong Hu, Weirong Gu

**Affiliations:** Obstetrics & Gynecology Hospital of Fudan University, Shanghai Key Lab of Reproduction and Development, Shanghai Key Lab of Female Reproductive Endocrine Related Diseases, Shanghai, China

**Keywords:** preeclampsia, cellular senescence, Mendelian randomization, colocalization analysis, gene expression, DNA methylation

## Abstract

**Background:**

The causal role of cellular senescence in preeclampsia pathogenesis is not fully established. This study aimed to systematically prioritize key senescence-related genes potentially driving preeclampsia using a Mendelian randomization (MR) framework.

**Results:**

We integrated genome-wide association studies (GWAS) of preeclampsia with expression, methylation, and proteomic quantitative trait loci (eQTLs/mQTLs/pQTLs) data for 866 senescence-related genes. Summary-data-based MR (SMR) coupled with the HEIDI (Heterogeneity in Dependent Instruments) test were used to assess causal associations and pleiotropy. Colocalization analysis evaluated shared genetic variants between QTLs and preeclampsia GWAS signals. Significant MR findings were explored for replication in an independent GWAS cohort (GCST90301704). Preliminary experimental support involved RT-PCR analysis of candidate genes in placental tissues from 10 preeclampsia patients and 5 gestational age-matched (34–38 weeks) healthy controls. Integration of SMR/HEIDI tests and colocalization (PPH4 > 0.5) prioritized 12 eQTLs, 62 mQTLs, and 2 pQTLs linked to preeclampsia. mQTL-eQTL analysis implicated methylation-regulated expression of *ATG16L1*, *PMVK*, and *MAP3K14*, offering valuable hypotheses for mechanistic studies.

**Conclusion:**

Placental RT-PCR showed upregulated *ATG16L1* and downregulated *PMVK*, *MAP3K14*, *NSUN2*, and *CDC25A* in preeclampsia. Key genes (*ATG16L1*, *PMVK*, *MAP3K14*, *NSUN2*, *CDC25A*) link cellular senescence to preeclampsia, offering insights for mechanistic studies and therapeutic targeting.

## Introduction

Preeclampsia, a pregnancy-specific hypertensive disorder affecting 2-8% of pregnancies globally (1), is a major contributor to maternal mortality, accounting for approximately 18% of direct maternal deaths (2). Characterized by new-onset hypertension and potential damage to vital organs like the liver and kidneys (3), the prevalence and severity of preeclampsia underscore the urgent need to understand its underlying causes. Despite extensive research, the precise mechanisms driving its development remain elusive, hindering the development of effective early diagnostic tools and curative treatments beyond delivery (2).

Among the factors that may contribute to the pathogenesis of preeclampsia, cellular senescence has gained increasing attention. Characterized by permanent cell cycle arrest (4), cellular senescence has been implicated in various pregnancy-related complications, including preeclampsia (5). Abnormal expression of cellular senescence biomarkers is observed in the placentas and peripheral blood of preeclampsia patients. Moreover, elevated activity of cell cycle inhibitors not only induces cell cycle arrest but also promotes senescence (5), while reduced expression of senescence-suppressing factors further exacerbates the process (6). Furthermore, elevated circulating levels of senescence-associated secretory phenotype (SASP) factors (7), upregulated SASP gene expression in placentas from pregnancies complicated by preeclampsia and fetal growth restriction (FGR) (8), and links between placental lactate accumulation, histone lactylation, and premature trophoblast senescence (9) suggest that these interconnected biological processes may play a pathogenic role in the development of preeclampsia and FGR. However, much of the current evidence stems from observational or single-omics association studies, limiting the ability to establish causal relationships between specific senescence-related genes and preeclampsia development. Establishing causality is crucial for understanding disease mechanisms and identifying targeted interventions.

To address this challenge, our study employs a Mendelian Randomization (MR) framework, leveraging multi-omics data through the Summary-data-based Mendelian Randomization (SMR) approach. SMR integrates summary statistics from large-scale Genome-Wide Association Studies (GWAS) with molecular quantitative trait loci (QTL) data (e.g., expression QTLs - eQTLs, methylation QTLs - mQTLs, protein QTLs - pQTLs) to infer potential causal associations between molecular traits and disease risk (10). By using genetic variants robustly associated with molecular traits as instrumental variables (IVs), MR mimics a randomized controlled trial design, thereby minimizing biases from confounding and reverse causation that often affect observational studies. We incorporated the HEIDI (Heterogeneity in Dependent Instruments) test to detect potential horizontal pleiotropy (where a genetic variant affects the outcome via pathways independent of the exposure) (11) and colocalization analysis to assess whether identified associations between QTLs and preeclampsia GWAS signals are likely driven by shared causal variants, strengthening biological plausibility (12). This multidimensional analytical strategy overcomes the limitations of traditional single-omics research, offering a more comprehensive perspective for deciphering the molecular mechanisms of complex diseases.

Given the evidence linking cellular senescence to preeclampsia, we used a hypothesis-driven approach to deeply investigate this specific biological pathway. This method complements broader genome-wide screens by offering targeted insights into potential causal factors within the senescence network. Therefore, this study aims to systematically screen for and prioritize cellular senescence-associated genes with potential causal effects on odds of preeclampsia using the SMR methodology. By identifying potentially causal genes, our findings aim to provide a data-driven foundation of prioritized candidates for developing new biomarkers and therapeutic targets.

## Materials and methods

### Study design

**Figure 1:**
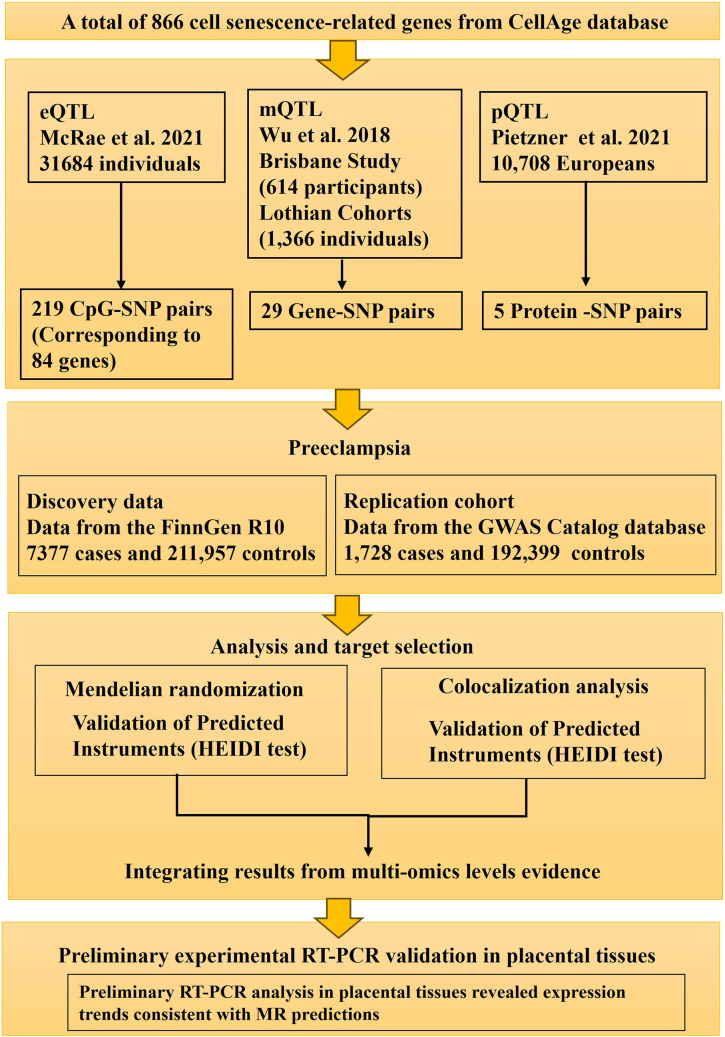
Study design flow chart. This diagram outlines the key stages of the study, including the selection of senescence-related genes, acquisition of GWAS summary statistics for preeclampsia and multi-omics QTL data (eQTLs, mQTLs, pQTLs), the Mendelian Randomization analysis pipeline (SMR, HEIDI, Colocalization), and subsequent experiments (replication in an independent GWAS cohort and differential expression analysis in placental tissues).

This study employed a multi-stage design to investigate the role of cellular senescence genes in preeclampsia. Initially, a comprehensive Mendelian Randomization (MR) analysis was conducted using publicly available summary-level data to identify potential causal links between genetically predicted molecular traits (gene expression, DNA methylation, protein abundance) related to 866 senescence genes and odds of preeclampsia. Subsequently, the expression of candidate genes prioritized from the MR findings were characterized using RT-PCR analysis of human placental tissues collected from preeclampsia patients and healthy controls. The MR component of the study was designed and reported following the Strengthening the Reporting of Observational Studies in Epidemiology using Mendelian Randomization (STROBE-MR) guidelines (13). The design and analytical strategy of the study are presented in **Figure 1**.

### Data sources

A total of 866 genes associated with cell senescence were sourced from the CellAge database (https://genomics.senescence.info/cells/) (14). For the identification of genetic variants associated with preeclampsia, we utilized summary GWAS data from two distinct cohorts. The discovery set was sourced from the FinnGen R10 cohort (finngen_R10_O15_PREECLAMPS), which included 7,377 cases and 211,957 controls of European ancestry (15). In the discovery cohort, preeclampsia was defined using the ICD-10 (International Statistical Classification of Diseases and Related Health Problems, 10th Revision) code O14. Validation was performed using dataset from the GWAS Catalog database (GCST90301704), comprising 1,728 cases and 192,399 controls of European ancestry, in which preeclampsia was defined as participants who self-reported having PE (code 1073 in Data-Field 20002) or had ICD-10 diagnostic codes O11, O12.0–O12.2, O13–O14.1, O14.9, and O15.0–O15.9 (16).

Blood eQTL summary data were obtained from the eQTLGen consortium, encompassing genetic and gene expression data from 31,684 individuals of European ancestry (17). Blood mQTL summary data were obtained from a meta-analysis of two European cohorts: the Brisbane Systems Genetics Study with 614 participants and the Lothian Birth Cohorts with 1,366 participants (11). Blood pQTL summary data were obtained from the study by Pietzner et al. (18), including data from 10,708 European individuals. The detailed information for all the datasets used in this study were listed in **Supplementary Table S1**.

We utilized tissue-specific expression eQTL data from the GTEx database (v8 release), covering 838 donors and 17,382 samples across 52 tissues and two cell lines (19). Given that placental tissue is unavailable in GTEx, the uterus was selected as the most relevant maternal tissue for this analysis. Uterine eQTL data can potentially reflect the influence of maternal genetic factors on preeclampsia via the uterine environment. Therefore, we focused on uterus tissue for tissue-specific analyses.

### SMR analysis

We employed the Summary-data-based Mendelian Randomization (SMR) method, implemented in the SMR software (v1.3.1), to test for causal associations between the blood QTLs (eQTLs, mQTLs, pQTLs) and preeclampsia (10). SMR uses the top *cis*-QTL associated with a molecular trait (gene expression, methylation probe, protein level) as an instrumental variable (IV). We selected the strongest *cis*-QTL (lowest two-sided P-value, P < 5 × 10^-8^) within a 1 Mb window ( ± 1,000 kb) of the target gene/probe as the primary IV for each trait. Statistical power was carried out using an online web tool (http://glimmer.rstudio.com/kn3in/mRnd).

The HEIDI (Heterogeneity in Dependent Instruments) test was applied to distinguish causality from linkage disequilibrium (LD)-induced correlation (linkage) or horizontal pleiotropy (11). The HEIDI test utilizes multiple SNPs in LD (0.05 < r² < 0.9) with the top *cis*-QTL, with LD estimated from the 1000 Genomes Project Phase 3 European population reference panel. Significant heterogeneity (P-HEIDI ≤ 0.01) suggests the association observed in SMR might be due to pleiotropy or linkage rather than a direct causal effect of the molecular trait on preeclampsia (20).

We also performed a multi-SNP SMR analysis, which aggregates association signals from multiple, largely independent *cis*-QTLs (P < 5 × 10^-8^, LD r² < 0.9 with the top SNP, if available) for a given molecular trait to potentially enhance statistical power (21). The SMR analysis leveraged high-quality summary statistics from source studies that had already undergone rigorous quality control, including filtering for imputation quality (INFO) and Hardy-Weinberg equilibrium (HWE). Within the SMR pipeline, we applied further standard filters: Associations were considered potentially causal if they met the following criteria: P-SMR < 0.05 (for the top IV), P-SMR_multi < 0.05 (if multi-SNP analysis applicable), and P-HEIDI > 0.01 (indicating no significant heterogeneity). Standard quality control filters were applied: SNPs with minor allele frequency (MAF) < 0.01 or ambiguous alleles were excluded. SNPs with allele frequency discrepancies > 0.2 between the GWAS and QTL datasets were removed, with a tolerance for up to 5% of SNPs having such discrepancies (22).

To explore potential regulatory mechanisms, we performed SMR analyses treating methylation levels (mQTLs) as the exposure and gene expression levels (eQTLs) as the outcome. We also investigated potential links between gene expression (eQTLs as exposure) and protein abundance (pQTLs as outcome) (23).

To account for the large number of tests, we also applied the Benjamini-Hochberg False Discovery Rate (FDR) correction within each omics layer. Given the exploratory nature of this study and the expected modest effect sizes, we used the nominal two-sided P-value for initial candidate screening but highlight the FDR results for context and interpret all findings with caution.

### Colocalization analysis

The colocalization analysis was conducted using the R package ‘coloc’ to evaluate the probability that cell senescence-related cis-QTLs (eQTLs, mQTLs, pQTLs) and preeclampsia GWAS signals share a common causal variant within a defined genomic region. Colocalization suggests that the genetic association with the disease phenotype might be mediated through the identified molecular trait (e.g., gene expression). The ‘coloc’ method calculates posterior probabilities (PP) for five hypotheses: H0 (no association with either trait), H1 (association only with trait 1 - QTL, H2 (association only with trait 2 - preeclampsia), H3 (association with both traits, distinct causal variants), and H4 (association with both traits, shared causal variant). Analysis was performed on SNPs within 1 Mb windows centered on the top cis-QTL. The prior probability P12 (prior probability of association with both traits) was set to 5 × 10^-5^ (24). Following common practice, a posterior probability PPH4 > 0.5 was considered suggestive evidence of colocalization, while PPH4 > 0.8 was considered as strong evidence of colocalization (25–28).

### Two-sample MR analysis

SNPs significantly associated with the levels of candidate eQTL and pQTL signals at a genome-wide level were screened with a threshold of P < 1 × 10^-5^. Then SNPs with a minor allele frequency (MAF) > 0.01 were selected. To reduce redundancy, linkage disequilibrium (LD) among SNPs was excluded according to R2 < 0.3 within a 500 kb window. Furthermore, F-statistics for these IVs were calculated using the formula F = R2*(N-2)/(1-R2), of which R2 represents the proportion of phenotypic variance explained by a single SNP and N refers to sample sizes (29). SNPs exhibiting an F-statistic less than 10 were considered to be poor IVs and were therefore excluded (29).

A two-sample MR analysis (using multiple independent SNPs) was executed between these curated IVs and odds of preeclampsia, which complements SMR analysis (typically using the top cis-SNP). We applied inverse variance weighting (IVW) (30), weighted median (31), weighted mode (32) and MR-Egger (33) to calculate the odds ratio and confidence interval, with IVW as the main approach. The findings were presented through scatter plots showcasing IV impacts on exposures and outcomes and forest plots which illustrate SNP effect estimates. All results were corrected for multiple testing using the Benjamini-Hochberg False Discovery Rate (FDR) method.

To exclude pleiotropy, MR-Egger was utilized, where pleiotropy was indicated if the intercept term is significant (34). Cochran’s Q was utilized for heterogeneity identification among IVs (35). Furthermore, MR-PRESSO and Radial MR were applied for outlier elimination and correcting for horizontal pleiotropy (34). In addition, Steiger tests were incorporated to examine causal directions (36). A leave-one-out approach was applied in the MR analysis, where each SNP was sequentially omitted to evaluate its individual impact on the overall causal inference. Funnel plots were also generated to showcase the publication biases. All analyses were executed in R version 4.3.1, utilizing the “Two-sample MR” and “RadialMR” package.

### Statistical software and visualization

All statistical analyses were conducted using R (v4.3.0). Manhattan plots were generated using “ggplot2”, and forest plots using “forestplot”. Locus and effect plots for SMR results were generated using scripts adapted from Zhu et al. (20) (SMRLocusPlot, SMREffectPlot).

### Expression profiles of key genes

Differential expression analysis was performed on publicly available GEO dataset GSE75010. Differential expression analysis was conducted using the “limma” package, with significance set at a Wilcoxon p-value < 0.05. Results were visualized using “ggplot2”. Expression patterns of key genes were then investigated and visualized in box plots. In addition, the clinical information was extracted from this dataset: age (maternal age), BMI, and delivery mode (including C-section and vaginal) to adjust the expressions of key genes against these variables. For this purpose, the R package glmnet was used. The results were presented in a forest plot.

### Human placental tissue collection

To assess the differential expressions of candidate genes prioritized by the MR analysis, placental tissue samples were collected immediately after delivery. The cohort included 10 patients diagnosed with preeclampsia (PE) and 5 normotensive control participants, matched for gestational age at delivery (34–38 weeks) and maternal age. Preeclampsia diagnosis adhered to the 2013 American College of Obstetricians and Gynecologists (ACOG) criteria. Control participants had uneventful, normotensive pregnancies. Exclusion criteria for both groups were: multiple gestations, known major fetal congenital or chromosomal abnormalities, pre-existing diabetes mellitus, significant chronic maternal diseases potentially impacting pregnancy (e.g., autoimmune disorders, chronic renal disease), and clinical evidence of chorioamnionitis. Ethical approval for sample collection and analysis was granted by the Ethics Committee of the Obstetrics and Gynecology Hospital of Fudan University (Ethics No.: 2022-115). Written informed consent was obtained from all participants prior to sample collection.

### Real-time PCR

Total RNA was extracted from placental tissues using Biozol Reagent (Bioer Technology), followed by DNase treatment (according to manufacturer’s protocol) to eliminate potential genomic DNA contamination. RNA quality and concentration were assessed using a Qubit 4.0 Fluorometer (Invitrogen) and visualized via agarose gel electrophoresis. Reverse transcription was performed using Hifair^®^ III Reverse Transcriptase (Yeasen Biotechnology) with 800 ng of total RNA input per reaction. qPCR reactions were conducted in triplicate using SYBR Green Master Mix (Yeasen) on a LightCycler 480 II system (Roche). Thermal cycling conditions were: initial denaturation at 95 °C for 2min, followed by 40 cycles of 95 °C for 10 s and 60 °C for 30 s. Primer sequences were designed for target genes (ATG16L1, PMVK, MAP3K14, NSUN2, CDC25A) and the reference gene *Beta actin* Amplification efficiency and specificity were confirmed using standard curves and melt curve analysis. Relative gene expression was calculated using the 2−ΔΔCt method. The primer sequences used were:

*ATG16L1* (Forward: AAGGAACCTCTACCAGTCGAACAG, Reverse: TTAGTGGCTGCTCTGCTGATGG);
*PMVK* (Forward: CTGTTCAGCGGCAAGAGGAAATC, Reverse: CGGAGGACAGCACAGACATCAG);
*MAP3K14* (Forward: CACAGGATGGAGGACAAGCAGAC, Reverse: ACAAAGGGACAATTCTGGGTGAGG);
*CDC25A* (Forward: TGAGGATGATGGCTTCGTG, Reverse: CGTTCTGGTCTCTTCAACACTG);
*NSUN2* (Forward: TCGTCCATCAAGCCAAGAG, Reverse: TTCTCATAGTGCCGTCTCCA);
*Beta actin* (Forward: GGCCAACCGCGAGAAGATGAC, Reverse: GGATAGCACAGCCTGGATAGCAAC).

### Statistical analysis for expression analysis

Statistical analyses for the RT-PCR data were performed using GraphPad Prism (Version 8.0.0). Differences in gene expression between the PE and control groups were assessed using the Mann - Whitney U test. A two-sided P-value < 0.05 was considered statistically significant.

### Ethical statement

The study protocol, including human participation and placental tissue collection, was approved by the Ethics Committee of the Obstetrics and Gynecology Hospital of Fudan University (Approval No.: 2022-115). Written informed consent was obtained from all participants before sample collection. The Mendelian Randomization analysis used publicly available, anonymized summary-level data from large-scale consortia and databases, thus not requiring separate ethical approval.

## Result

### Mendelian randomization analyses linking blood molecular QTLs to odds of preeclampsia

#### Putative causal effects of blood gene expression (eQTLs) on preeclampsia

**Figure 2:**
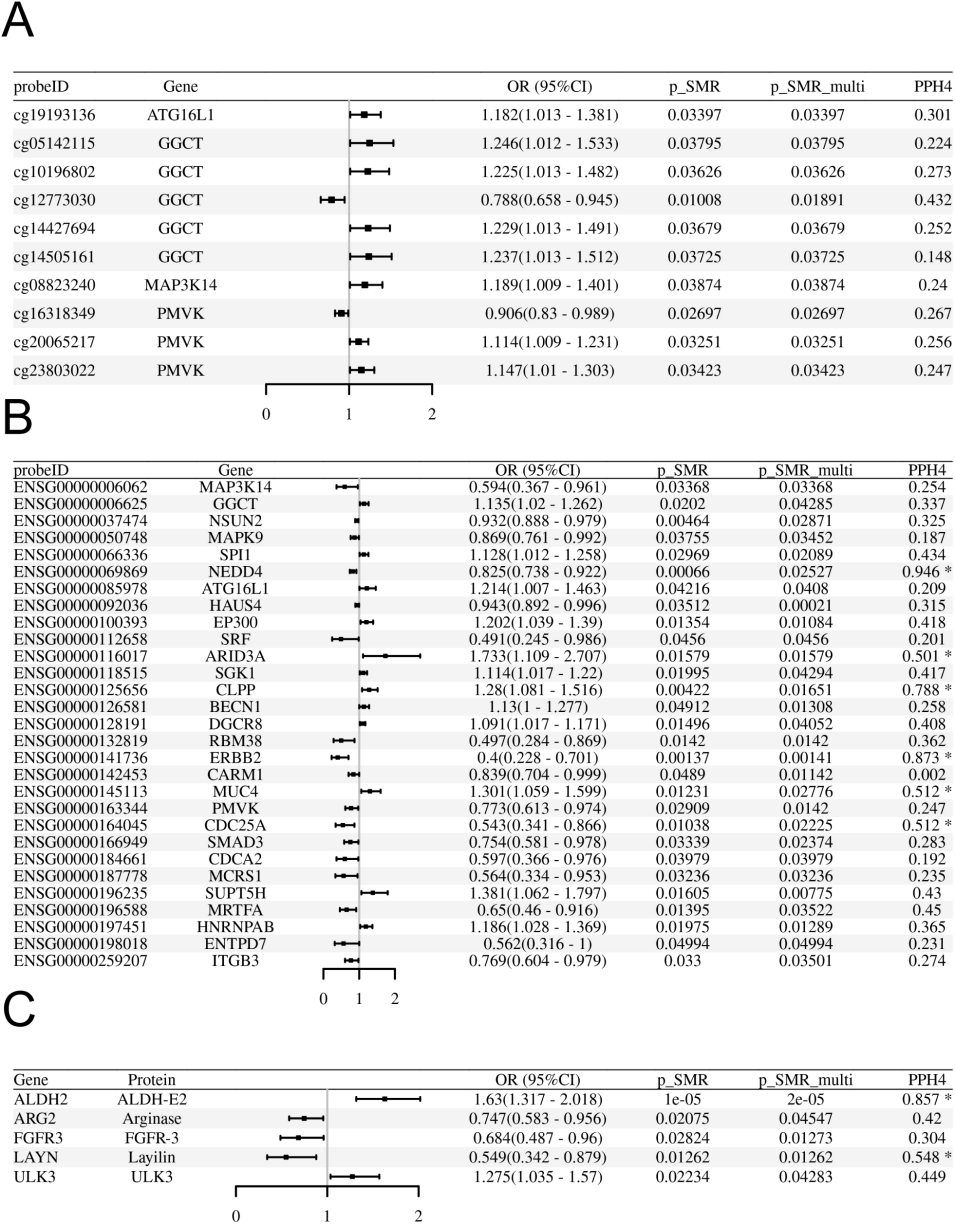
SMR analyses linking blood molecular QTLs to odds of preeclampsia in the FinnGen R10 cohort. **(A-C)** Forest plots display the Odds Ratio (OR) and 95% Confidence Interval (CI) for each significant association passing SMR (P<0.05) and HEIDI (P>0.01) tests. The x-axis represents the OR for preeclampsia per standard deviation increase in the molecular trait level. An OR > 1 indicates increased risk, while OR < 1 indicates decreased risk. **(A)** Associations for significant blood methylation QTLs (mQTLs). **(B)** Associations for significant blood expression QTLs (eQTLs). **(C)** Associations for significant blood protein QTLs (pQTLs).

Using SMR analysis with HEIDI filtering (P-SMR < 0.05, P-SMR_multi < 0.05, P-HEIDI > 0.01), we identified 29 senescence-related genes whose genetically predicted expression levels in blood were putatively associated with odds of preeclampsia (**Figure 2A**; **Supplementary Table S2**). Higher predicted expression of 12 genes (including *ATG16L1*, *BECN1*, *EP300*, *SGK1*) was associated with increased odds of preeclampsia, while higher predicted expression of 17 genes was associated with decreased risk (**Figure 2B**). Specifically, the expression of *ATG16L1* was positively associated with odds of preeclampsia (OR: 1.214, 95%CI=1.007-1.463, P-SMR=0.042, FDR-SMR=0.644) Subsequent colocalization analysis provided suggestive evidence (PPH4 > 0.5) that the association signals for 6 of these 29 genes likely share a common causal variant with the preeclampsia GWAS signal (**Figure 2A**). Under a stringent threshold (PPH4 > 0.8), only 2 genes (*NEDD4* and *ERBB2*) showed strong colocalization evidence (**Figure 2A**). It’s worth noting that after applying a Benjamin Hochberg correction for multiple testing, no associations met the strict FDR threshold (FDR-SMR < 0.05) (**Supplementary Table S2**). Given the hypothesis generating nature of our study, we proceeded to prioritize candidates based on the convergence of nominal significance signals and other supporting evidence.

#### Putative causal effects of blood DNA methylation (mQTLs) on preeclampsia

Applying the same SMR/HEIDI criteria, we identified 219 methylation CpG sites (mapping to 84 unique senescence-related genes) significantly associated with odds of preeclampsia (**Figure 2B**; **Supplementary Table S3**). Colocalization analysis indicated suggestive evidence (PPH4 > 0.5) for shared causal variants at 62 of these CpG sites (corresponding to 27 unique genes) and strong evidence (PPH4 > 0.8) for 25 of these CpG sites (corresponding to 12 unique genes) (**Figure 2B**). No associations met the strict FDR threshold (FDR-SMR < 0.05) (**Supplementary Table S3**).

#### Putative causal effects of blood protein abundance (pQTLs) on preeclampsia

SMR/HEIDI analysis identified 5 senescence-related genes whose genetically predicted protein abundance levels in blood were associated with odds of preeclampsia (**Figure 2C**; **Supplementary Table S4**). Colocalization analysis provided suggestive colocalization evidence (PPH4 > 0.5) for 2 of these proteins (ALDH2 and LAYN) and strong evidence (PPH4 > 0.8) for ALDH2 (**Figures 2C, F**). No associations met the strict FDR threshold (FDR SMR < 0.05) (**Supplementary Table S4**).

### Investigating regulatory effects: mQTL-eQTL integration analysis

We investigated whether genes identified in both the mQTL and eQTL analyses showed evidence of methylation influencing expression in the context of preeclampsia. Comparing the significant gene lists, 8 genes were found to be associated with odds of preeclampsia at both the methylation (via specific CpG sites) and expression levels (**Table 1**).

**Table 1:** SMR analysis results for methylation levels (exposure) influencing gene expression (outcome).

ExPo-ID	Outco-Gene	Symbol	P-SMR	P-SMR-multi	OR-SMR	95% Cl-SMR	OR (95% CI) per 1 SD increase
cg19193136	ENSG00000085978	*ATG16L1*	4.23E-11	4.23E-11	2.159	1.718 - 2.715	2.159 (1.718 - 2.715)
cg08823240	ENSG00000006062	*MAP3K14*	2.55E-07	2.55E-07	0.717	0.632 - 0.814	0.717 (0.632 - 0.814)
cg16318349	ENSG00000163344	*PMVK*	1.92E-23	6.33E-22	1.446	1.345 - 1.555	1.446 (1.345 - 1.555)

Further SMR analysis treating methylation as the exposure and expression as the outcome (P-SMR < 0.05, P-SMR_multi < 0.05, P-HEIDI > 0.01) identified 3 genes (*ATG16L1*, *MAP3K14*, *PMVK*) where specific CpG methylation levels appeared to causally influence gene expression levels (**Table 1**; **Supplementary Table S5**), and importantly, both the CpG site and the gene’s expression were associated with odds of preeclampsia in the primary analyses.

**Figure 3:**
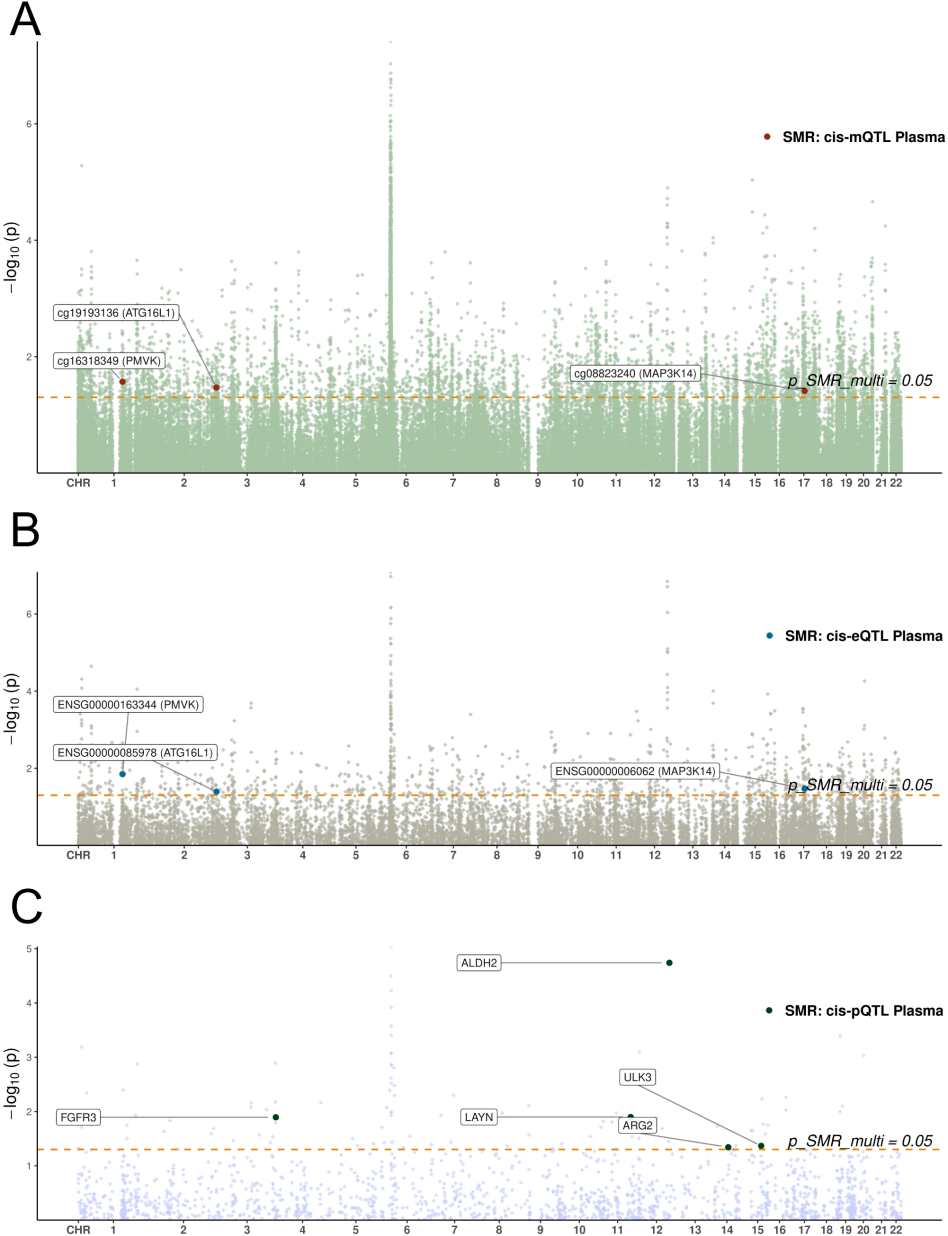
Manhattan plots of SMR analyses linking blood molecular QTLs to preeclampsia. **(A-C)** Manhattan plots display the chromosomal distribution and statistical significance (-log10 P-value) of SMR associations. The x-axis represents chromosomal position, and the y-axis represents the -log10(P-value) from the SMR test. The horizontal dashed line indicates the nominal significance threshold (P=0.05). Each point represents a tested molecular trait. **(A)** SMR results for blood cis-mQTLs. **(B)** SMR results for blood cis-eQTLs. **(C)** SMR results for blood cis-pQTLs.

Among the potential mechanisms involving notable genes, the following are identified (**Figure 3**): Methylation at cg19193136 positively associated with *ATG16L1* expression and odds of preeclampsia (**Supplementary Figure S1**); methylation at cg16318349 positively associated with *PMVK* expression but negatively with odds of preeclampsia (**Supplementary Figure S2**); methylation at cg08823240 positively associated with odds of preeclampsia but negatively with *MAP3K14* expression (**Supplementary Figure S3**). The location and annotation of these specific CpG loci were provided in **Supplementary Table S6**.

### Replication analyses

#### Tissue context: SMR analysis using uterine eQTLs

To assess relevance in a more proximate maternal tissue, we performed SMR analysis using GTEx uterine eQTL data. This analysis supported associations (P-SMR < 0.05, P-SMR_multi < 0.05, P-HEIDI > 0.01) for *CDC25A* (OR = 0.938, 95% CI: 0.892-0.987; **Supplementary Figures S4A, B**) and *NSUN2* (OR = 0.929, 95% CI: 0.877-0.985; **Supplementary Figures S4C, D**) expression with decreased odds of preeclampsia (**Supplementary Table S7**). Notably, the associations for both genes were also significant in the blood eQTL analysis, and the *CDC25A* signal showed suggestive colocalization evidence (PPH4 > 0.5) in blood.

#### Replication analysis in an independent preeclampsia GWAS (GCST90301704)

Replication attempts in the independent GCST90301704 GWAS cohort yielded limited support for the discovery findings, possibly due to far fewer sample sizes (n=1728) of this replication cohort than the discovery cohort (n=7377). Specifically, it showed that the power values of the top SNPs for these five candidates in the replication set were all relatively low, thereby explaining the poor validation efficiency in the replication set (**Supplementary Table S8**).

**eQTLs**: Only the association for higher *HAUS4* expression (OR = 1.152, 95% CI: 1.046-1.269) with increased risk was replicated (**Supplementary Table S9**), although this locus lacked colocalization evidence in the FinnGen discovery analysis.
**mQTLs**: Nine CpG sites (mapping to genes *AXL*, CDKN1A, KNDC1, SOX5) showed significant associations in the replication cohort (**Supplementary Table S10**); however, none of these replicated CpG sites corresponded to the genes identified in the integrated mQTL-eQTL analysis (*ATG16L1*, *MAP3K14*, *PMVK*, *CARM1*) or those highlighted in the uterine analysis (*CDC25A*, *NSUN2*).
**pQTLs**: No significant pQTL associations were replicated (**Supplementary Table S11**).

#### Causal associations between the expression levels of candidate genes and odds of preeclampsia

**Figure 4:**
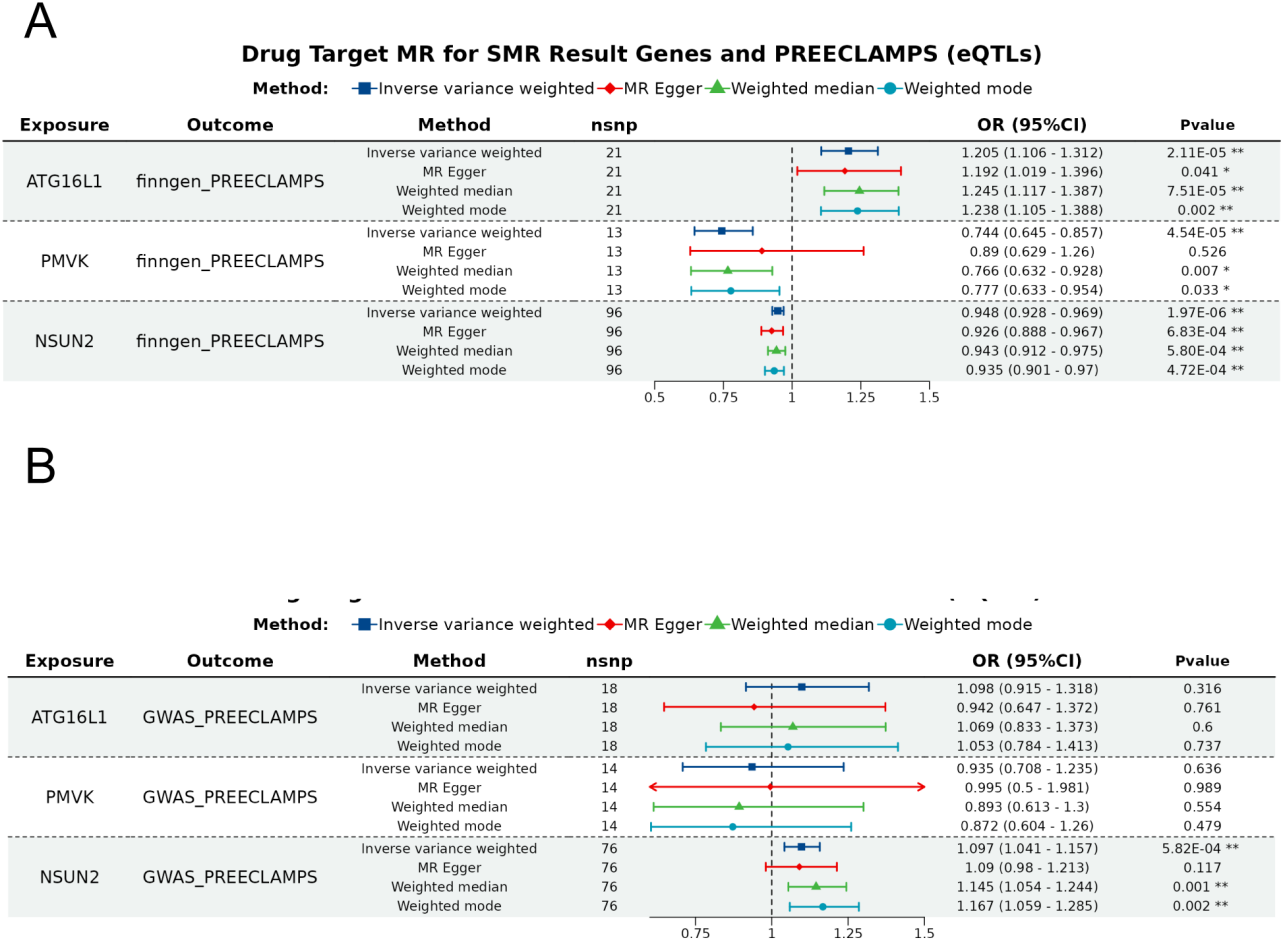
Forest plot of the SMR analysis results. The forest plots for the significant e/pQTLs and odds of preeclampsia in the **(A)** discovery dataset and **(B)** replication dataset. Different color denote distinct MR analysis methods, with * indicating P < 0.05 and ** indicating P < 0.001.

To ensure robustness, we performed Radial MR analyses using IVW and Egger models, identifying outliers by their heterogeneity contributions. After outliers removal, the expressions of *ATG16L1*, *PMVK* and *NSUN2* were found to be associated with odds of preeclampsia via the IVW method (**Figure 4A**; **Supplementary Table S12**). However, none of these signals were confirmed in the replication dataset, with the only significant signal NSUN2 exhibiting an inverse association with preeclampsia (**Figure 4B**, **Supplementary Table S12**). After the removal of these outlier SNPs, sensitivity analysis revealed no heterogeneity among these associations (**Supplementary Table S12**). Pleiotropy was observed in the association between *NSUN2*, *ATG16L1* and preeclampsia in the replication dataset as suggested by MR-Egger analysis (**Supplementary Table S12**). In addition, MR-PRESSO suggested potential pleiotropy for these association (**Supplementary Table S12**). Therefore, the causal associations between these factors should be interpreted with caution. The Steiger test confirmed the consistency in causal directions of these five signals on preeclampsia (**Supplementary Table S13**).

#### Prioritization of candidate genes

To select the most promising candidates from the numerous significant associations, we implemented a prioritization strategy based on the convergence of evidence. We prioritized genes that met one or more of the following criteria: (1) strong evidence of a regulatory mechanism from our mQTL-eQTL integration analysis, where methylation levels appeared to causally influence gene expression, and both were linked to preeclampsia; (2) evidence of a potential causal role in a more disease-relevant maternal tissue (uterus); and (3) supporting evidence (PPH4>0.5) from colocalization analysis. This led to the selection of five key genes: *ATG16L1*, *PMVK*, and *MAP3K14* were prioritized from the mQTL-eQTL analysis, while *CDC25A* and *NSUN2* were prioritized based on the significant findings in the uterine eQTL analysis, with *CDC25A* also showing colocalization support in blood.

#### Differential expression profile in placental tissues

**Figure 5:**
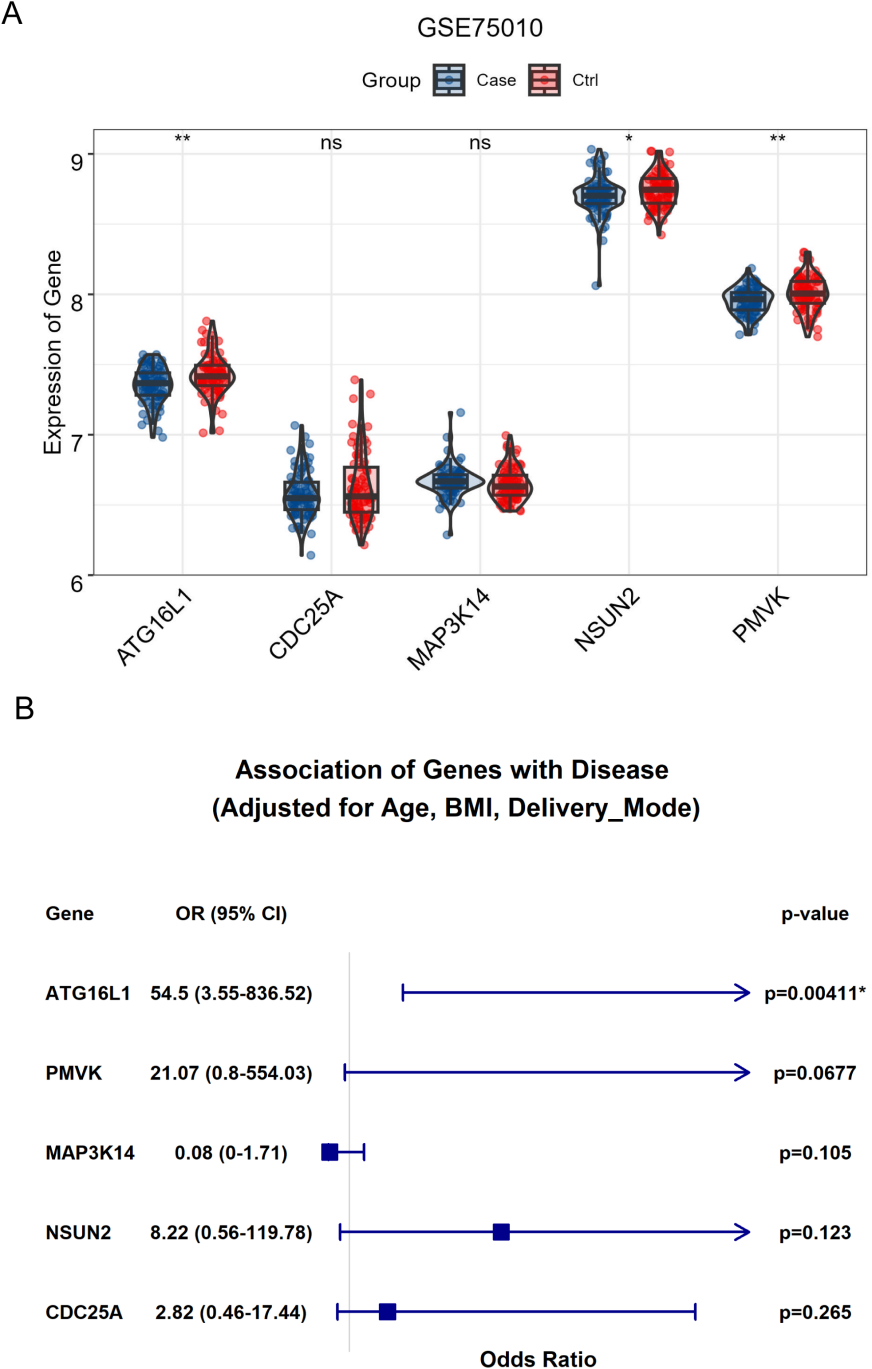
The significance of key genes in preeclampsia. **(A)** The bar plot demonstrated the significant differential expression for *ATG16L1*, *NSUN2* and *PMVK* in placental tissues between healthy control and patients with preeclampsia. **(B)** The association between the expression for *ATG16L1*, *NSUN2*, *PMVK* and preeclampsia after adjusting for variables including maternal age, BMI and delivery mode. * indicates significant associations; ** indicates a p-value < 0.01.

To further investigate the clinical relevance of key genes, differential expression analysis was conducted on RNA-seq data from the GEO dataset (GSE75010). The results revealed that the expressions of *ATG16L1*, *NSUN2* and *PMVK* were significantly downregulated in placental tissues from preeclampsia patients, whereas other key genes such as *CDC25A* and *MAP3K14* did not vary significantly between groups (**Figure 5A**). It’s worth noting that even after adjustment for variables including maternal age, BMI and delivery mode, the expression of ATG16L1 remained significantly positively associated with preeclampsia (**Figure 5B**).

#### Expression profiles of candidate genes in placenta

**Figure 6:**
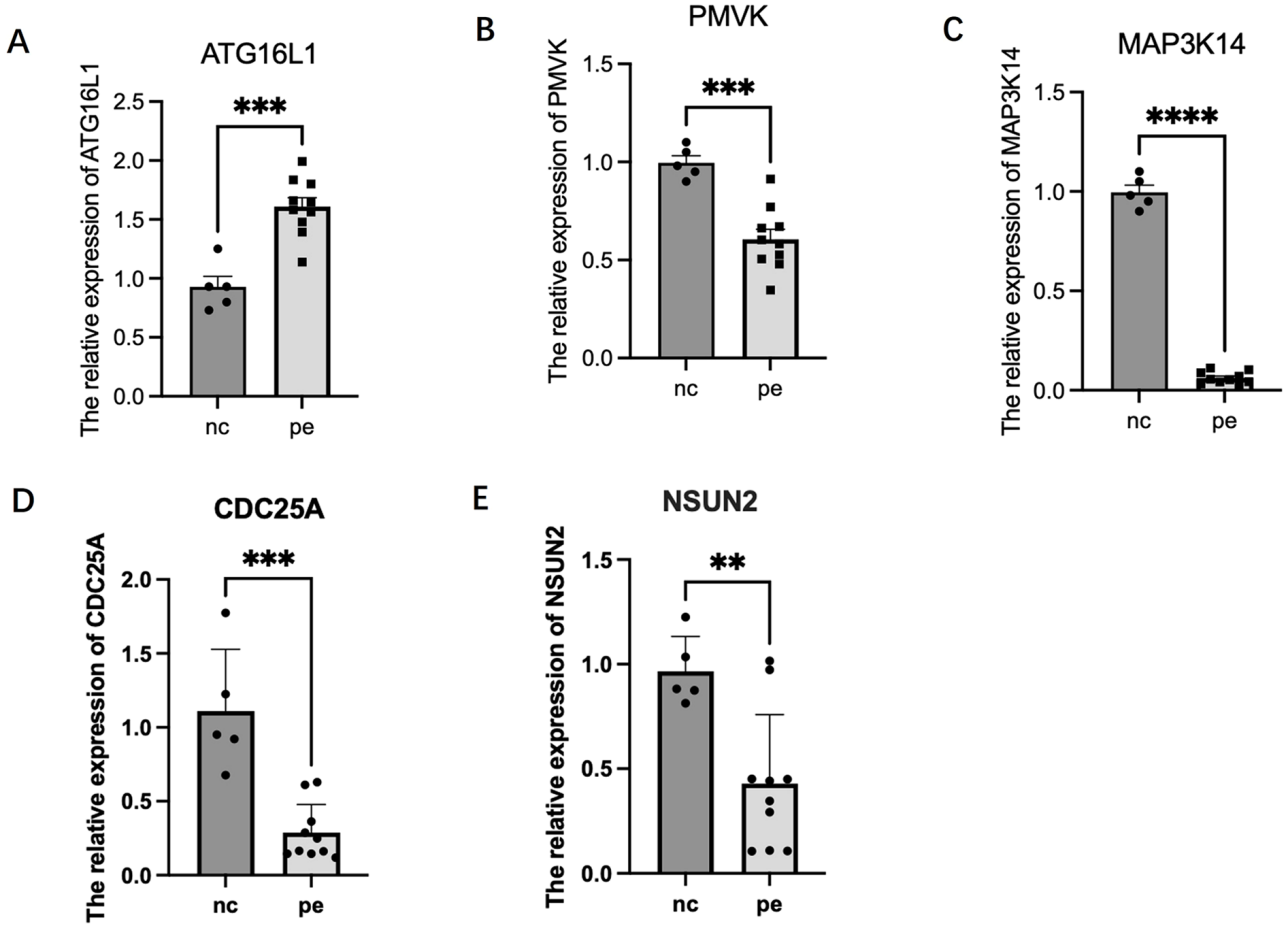
Relative mRNA expression levels of candidate senescence-related genes in human placental tissues. Gene expression was measured by RT-PCR in samples from patients with preeclampsia (PE, n=10) and gestational age-matched normotensive controls (Control, n=5). Expression levels were normalized to the reference gene ACTB (Beta-actin) and calculated using the 2^-ΔΔCt^ method, presented as fold change relative to the control group mean. Bars represent the mean ± Standard Error of the Mean (SEM). Statistical significance between groups was assessed using the Mann-Whitney U test. **P < 0.01; ***P < 0.001; ****P < 0.0001. **(A)** *ATG16L1*. **(B)** *PMVK*. **(C)** *MAP3K14*. **(D)** *CDC25A*. **(E)** *NSUN2*.

Preliminary RT-PCR analysis was performed on placental tissues from 10 PE patients and 5 gestational-age matched controls (**Figure 6**). The results suggested differential expression patterns for the prioritized candidate genes, largely aligning with SMR predictions, although constrained by the small control group size. *ATG16L1* expression appeared upregulated in PE placentas (P < 0.001), consistent with the SMR prediction linking higher expression to increased risk. *PMVK* (P < 0.001), *MAP3K14* (P < 0.0001), *NSUN2* (P < 0.01), and *CDC25A* (P < 0.001) expression levels appeared downregulated in PE placentas. These trends are consistent with the protective roles inferred from the mQTL-eQTL SMR analysis (for *PMVK*, *MAP3K14*) or the uterine eQTL analysis (for *NSUN2*, *CDC25A*). These preliminary experimental findings lend support to the potential relevance of *ATG16L1*, *PMVK*, *MAP3K14*, *NSUN2*, and *CDC25A* dysregulation in preeclampsia pathophysiology.

## Discussion

This study employed a multi-omics Mendelian Randomization approach to investigate the potential causal roles of cellular senescence-related genes in preeclampsia pathogenesis. Our findings implicate several genes operating through diverse pathways—including *ATG16L1*, *MAP3K14*, *PMVK*, *CDC25A*, and *NSUN2*—as potential mediators linking senescence processes to odds of preeclampsia. These genetic associations were further explored through tissue-specific MR analysis and RT-PCR in placental tissue.

Our MR analyses highlighted genes central to cellular stress and response pathways. Higher genetically predicted *ATG16L1* expression, a key autophagy gene (37), was linked to increased odds of preeclampsia. While autophagy is often protective, its dysregulation (either insufficient or excessive) is implicated in placental pathology (38), potentially impairing trophoblast function or modulating senescence-associated inflammation (39, 40). This aligns with our preliminary RT-PCR results, which suggested *ATG16L1* upregulation in preeclamptic placentas compared to controls. For *MAP3K14*, involved in NF-κB signaling and inflammation (41), lower genetically predicted expression was associated with increased risk. This finding, potentially supported by the observed trend of *MAP3K14* downregulation in our placental samples, hints that impaired NF-κB signaling or altered inflammatory resolution might contribute to preeclampsia, possibly intersecting with SASP regulation (7, 8). The mevalonate pathway gene *PMVK* also emerged, with higher genetically predicted expression (linked to higher methylation at cg16318349) associated with lower odds of preeclampsia. Our RT-PCR analysis showed a trend towards *PMVK* downregulation in PE placentas, consistent with the MR prediction that lower expression confers higher risk. While some studies link *PMVK* downregulation to p53 activation and cell cycle arrest (42), its precise role in the placenta, potentially involving p53-independent metabolic or signaling functions or specific effects of cg16318349 methylation, warrants further investigation to resolve this apparent complexity. While the differential expression analysis does not confirm causality, it demonstrates that the gene dysregulation predicted by our genetic analysis is present at the tissue level in the disease state, thus providing important downstream support for its potential involvement.

It’s worth noting that the expressions of 2 genes (*NEDD4* and *ERBB2*) showed strong colocalization evidence under a stringent threshold (PPH4 > 0.8). The *NEDD4* gene encodes an E3 ubiquitin protein ligase. It plays a crucial role in various cellular processes, including protein degradation, cell signaling, cell differentiation, and cell cycle regulation (15021885). In the context of preeclampsia, NEDD4 is involved in the regulation of trophoblast necrosis by mediating the ubiquitination of TAK1, thereby affecting placental development and function (43). Additionally, the NEDD4L gene is associated with hypertension and epithelial sodium transport (44). Given that preeclampsia is a pregnancy-specific hypertensive disorder, the involvement of NEDD4L in hypertension suggests a potential link between NEDD4 and preeclampsia. On the other hand, the *ERBB2* gene encodes a receptor tyrosine kinase and is a member of the epidermal growth factor receptor family. However, there is limited direct research on the relationship between the *ERBB2* gene and preeclampsia. Given its role in cell growth and differentiation, *ERBB2* may indirectly influence placental development and function, which are critical factors in the pathogenesis of preeclampsia.

Using uterine eQTL data as a proxy for maternal contributions, we found that higher genetically predicted expression of *CDC25A* and *NSUN2* was associated with decreased odds of preeclampsia. *CDC25A* regulates cell cycle transitions, and its dysregulation is linked to senescence (45); a previous MR study also hinted at its involvement in preeclampsia (46). *NSUN2*, an RNA methyltransferase, adds to growing evidence implicating RNA modifications in placental development and function, such as decidualization (47, 48). The observed trends of *CDC25A* and *NSUN2* downregulation in our preliminary RT-PCR analysis of preeclamptic placentas align with these protective MR associations. Collectively, these findings suggest that disruptions in placental cell cycle control (*CDC25A*) and RNA modification pathways (*NSUN2*) may contribute significantly to preeclampsia pathology.

Synthesizing these results, dysregulation across interconnected pathways—autophagy (*ATG16L1*), inflammation (*MAP3K14*), metabolism (*PMVK*), cell cycle (*CDC25A*), and RNA methylation (*NSUN2*)—appears to converge on promoting placental cellular senescence, contributing to preeclampsia pathophysiology. Senescence, particularly in trophoblasts, can impair placentation via multiple routes, including SASP-mediated inflammation disrupting spiral artery remodeling and causing endothelial dysfunction (49), and intrinsic impairment of trophoblast proliferation, migration, and differentiation. Connecting these senescence-associated genes and pathways to the broader pathophysiology of preeclampsia highlights potential cross-talk with these core mechanisms. For instance, the SASP can exacerbate systemic inflammation and endothelial dysfunction, key features of preeclampsia. Additionally, metabolic alterations (PMVK) may contribute to oxidative stress, further impairing placental function. Investigating functional links between these senescence-related genes and established preeclampsia susceptibility loci (e.g., near FLT1 (17)) represents an important future direction.

The identification of these candidate genes and pathways offers potential avenues for clinical translation, such as novel biomarkers (e.g., methylation patterns, circulating RNAs, SASP components) or therapeutic targets. However, considerable hurdles remain. Biomarker validation requires large, diverse prospective cohorts to establish sensitivity, specificity, and clinical utility. Therapeutic modulation of these pathways (e.g., using autophagy modulators, anti-inflammatories, senolytics) faces significant challenges regarding efficacy and, critically, safety during pregnancy, necessitating rigorous preclinical evaluation.

A key strength of our approach was the integration across multiple omics levels. The analysis identified CpG methylation sites apparently influencing gene expression, where both methylation and expression were associated with odds of preeclampsia, provides compelling candidates for epigenetic regulation in the disease. For *ATG16L1*, higher methylation at cg19193136 was unexpectedly associated with both higher gene expression and increased odds of preeclampsia. Conversely, for *MAP3K14*, higher methylation at cg08823240 was linked to lower expression but higher disease risk, aligning more closely with canonical gene silencing. The *PMVK* association (higher methylation linked to higher expression and lower risk) also deviated from simple promoter-silencing models. These complex methylation-expression relationships, particularly the positive correlations, underscore the need for further investigation (50). Potential mechanisms include methylation within gene bodies or enhancer regions affecting regulatory element binding, the influence of 5-hydroxymethylcytosine (5hmC) (51). Methylation in enhancer regions can either promote or stabilize the binding of transcriptional activators, thereby positively influencing gene expression (52). Additionally, 5hmC, which is often associated with active gene expression, may contribute to the regulation of *ATG16L1* expression through distinct mechanisms compared to traditional 5-methylcytosine. Elucidating these precise epigenetic mechanisms requires targeted studies mapping CpG locations relative to functional elements.

Several limitations must be acknowledged. Firstly, our SMR findings showed limited replication in the independent GCST90301704 cohort. While this could be due to insufficient power in the replication sample or false positives in our discovery analysis, it may also reflect the significant clinical and etiological heterogeneity of preeclampsia. Our discovery cohort (FinnGen) used a broad, EHR-based definition of preeclampsia, whereas the replication cohort employed more stringent clinical criteria. This difference could lead to discordance if the identified genes are associated only with specific subtypes of the disease. This highlights the challenge of replicating genetic associations for complex, heterogeneous syndromes. Secondly, the RT-PCR experiments had a small sample size, particularly in the control group (n=5), which limits the statistical power and reliability of the findings. This increases the risk of both Type I (false positives) and Type II (false negatives) errors. Although the observed expression trends for our candidate genes are consistent with our MR predictions, these results are preliminary and must be interpreted cautiously. Thirdly, a major limitation is the use of blood and uterine QTL data as proxies for the placenta. This tissue-context mismatch could significantly impact causal inference, as we may have missed true causal genes regulated by placenta-specific QTLs that are inactive in blood and uterus. Conversely, an observed association could be misleading if a genetic variant affects gene expression differently in the placenta compared to the proxy tissues. Future research must prioritize placenta-specific multi-omics QTL datasets to validate and build upon our findings. Fourthly, our study found a discrepancy between significant SMR results and non-significant colocalization results for top candidate genes like *ATG16L1* and *CDC25A*. This highlights the complementary nature of these methods. SMR tests for causal associations, while colocalization assesses shared causal variants. The lack of colocalization does not invalidate SMR findings but suggests a complex genetic architecture, such as causality driven by different variants in high LD. Thus, we consider SMR results as primary evidence for a causal link, acknowledging that further fine-mapping and functional studies using placenta-specific data are needed to elucidate the precise mechanisms. Fifthly, a major limitation is the lack of statistical significance after correction for multiple testing. This is common in under-powered MR omics studies. As a result, the aims of this study should be generating hypothesis, requiring substantial validation. Sixthly, sample overlap between exposure and outcome populations can potentially bias MR estimates. However, since our primary instruments were strong (F > 10), this bias is expected to be minimal. Finally, standard MR limitations apply, including the possibility of residual confounding or pleiotropy despite statistical tests (SMR/HEIDI, colocalization) and the reduced generalizability due to the primary reliance on European ancestry datasets.

Future research should prioritize validating these findings in larger, multi-ethnic and subtypically graded meta analyses and utilizing placenta-specific multi-omics QTL data when available. Prospective studies correlating biomarkers derived from these pathways with pregnancy outcomes are needed. Combining data from cohorts with detailed clinical information will be essential to explore the specific genetic architecture of different preconceptions. Ultimately, functional studies in relevant cell and animal models are essential to confirm the causal impact of *ATG16L1*, *PMVK*, *MAP3K14*, *NSUN2*, and *CDC25A* on senescence phenotypes and preeclampsia pathogenesis.

In conclusion, this multi-omics MR study, combined with preliminary experimental insights, pinpoints *ATG16L1*, *PMVK*, *MAP3K14*, *NSUN2*, and *CDC25A* as key candidate genes potentially mediating the link between cellular senescence pathways and odds of preeclampsia. While requiring further validation, these findings provide a valuable foundation for future research into the molecular mechanisms of preeclampsia and the development of targeted interventions.

## Data Availability

The original contributions presented in the study are included in the article/**Supplementary Material**. Further inquiries can be directed to the corresponding author/s.

## Supplementary material

Supplementary Figure 1 SMRLocusPlots and SMREffectPlots for *ATG16L1*. **(A)** SMR Effect Plot (eQTL); **(B)** SMR Effect Plot (mQTL at cg19193136); **(C)** SMR Locus Plot (eQTL); **(D)** SMR Locus Plot (mQTL at cg19193136).

Supplementary Figure 2 SMRLocusPlots and SMREffectPlots for *PMVK*. **(A)** SMR Effect Plot (eQTL); **(B)** SMR Effect Plot (mQTL at cg16318349); **(C)** SMR Locus Plot (eQTL); **(D)** SMR Locus Plot (mQTL at cg16318349).

Supplementary Figure 3 SMRLocusPlots and SMREffectPlots for *MAP3K14*. **(A)** SMR Effect Plot (eQTL); **(B)** SMR Effect Plot (mQTL at cg08823240); **(C)** SMR Locus Plot (eQTL); **(D)** SMR Locus Plot (mQTL at cg08823240).

Supplementary Figure 4 SMRLocusPlot and SMREffectPlot for *CDC25A*, and *NSUN2*. **(A)** SMR Effect Plot of *CDC25A* in eQTL; **(B)** The SMR locus plot for *CDC25A* in eQTL; **(C)** SMR Effect Plot for *NSUN2* in eQTL; **(D)** The SMR locus plot for *NSUN2* at cg08823240 in eQTL.
