# Supplementary figures and images for: Investigating the causal role of cellular senescence-related genes in preeclampsia: a multi-omics Mendelian randomization study with differential expression analysis
Source: Front Endocrinol (Lausanne). 2025 Oct 27;16:1661666. doi: 10.3389/fendo.2025.1661666 (PMC12597756; doi:10.3389/fendo.2025.1661666)

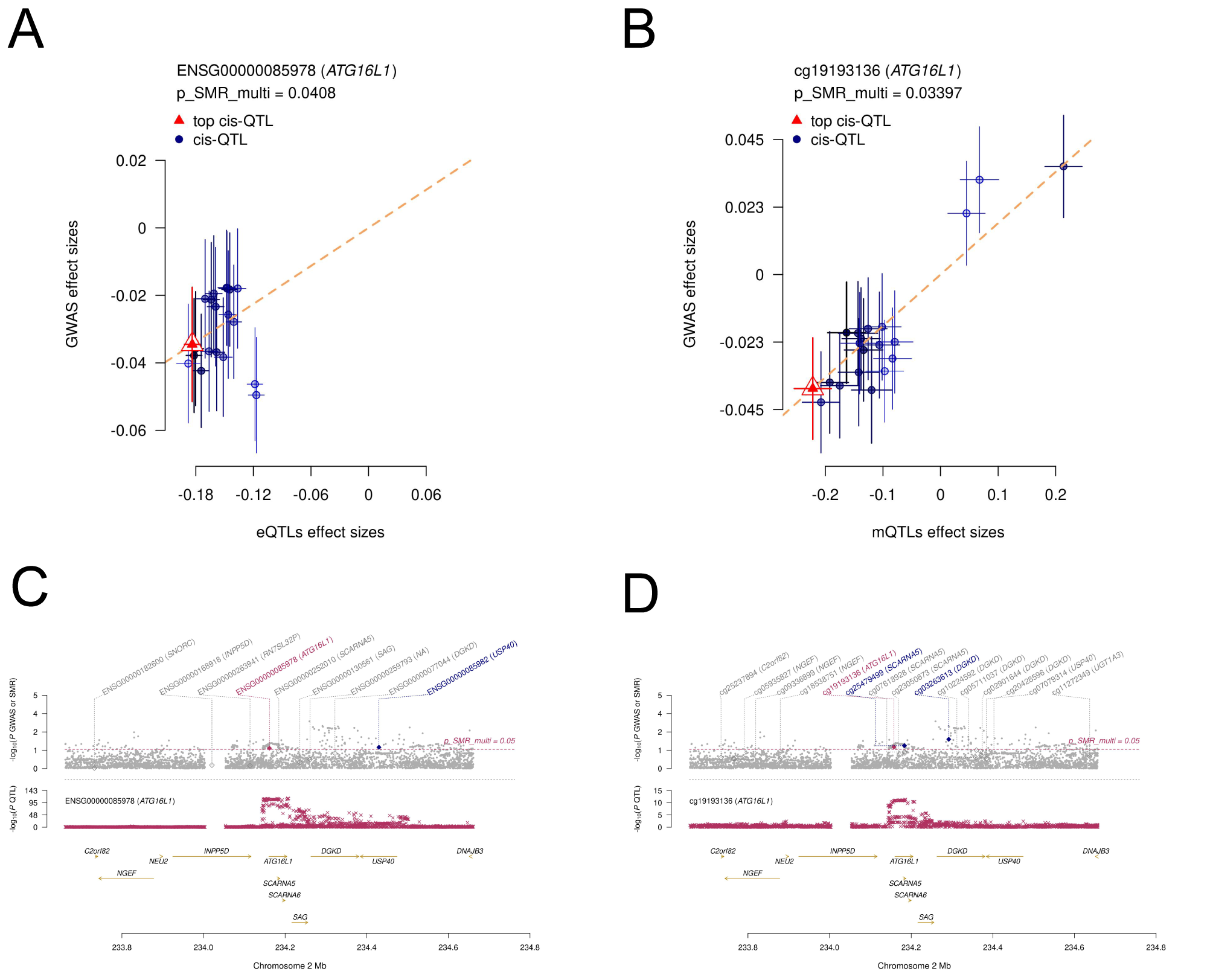

Supplement: Supplementary Figure 1 — SMRLocusPlots and SMREffectPlots for ATG16L1. (A) SMR Effect Plot (eQTL); (B) SMR Effect Plot (mQTL at cg19193136); (C) SMR Locus Plot (eQTL); (D) SMR Locus Plot (mQTL at cg19193136). [file Image1.tif]

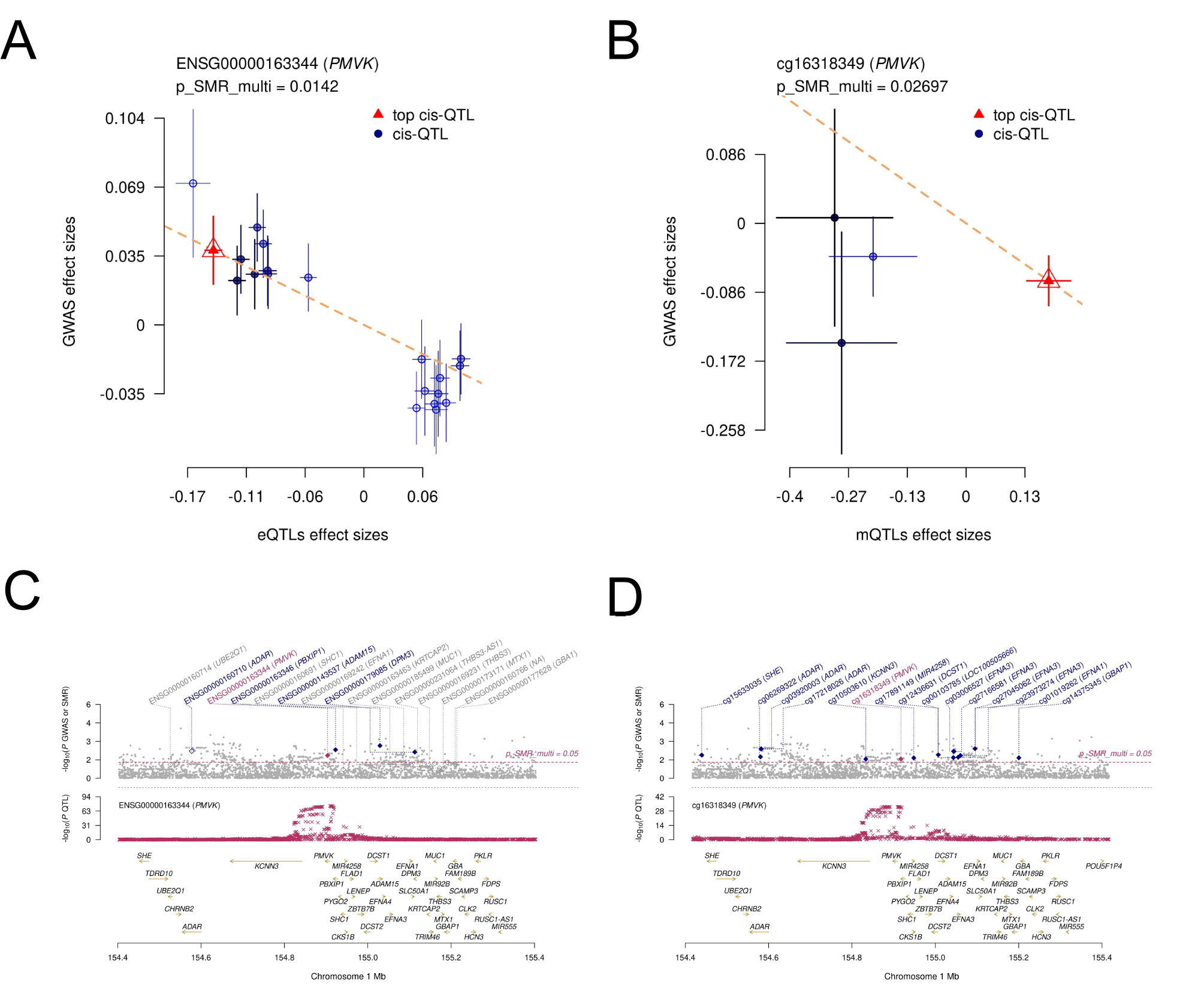

Supplement: Supplementary Figure 2 — SMRLocusPlots and SMREffectPlots for PMVK. (A) SMR Effect Plot (eQTL); (B) SMR Effect Plot (mQTL at cg16318349); (C) SMR Locus Plot (eQTL); (D) SMR Locus Plot (mQTL at cg16318349). [file Image2.tif]

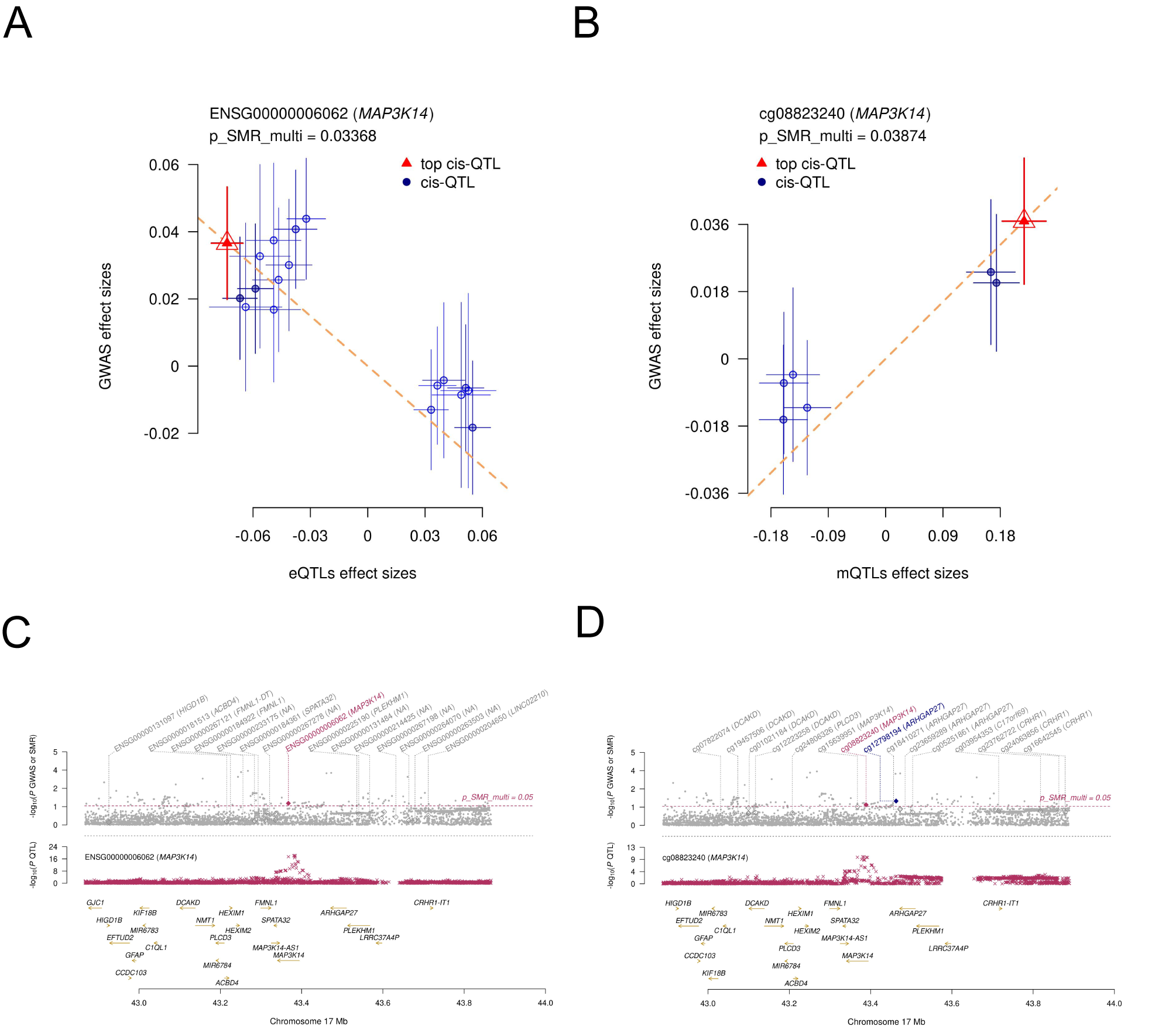

Supplement: Supplementary Figure 3 — SMRLocusPlots and SMREffectPlots for MAP3K14. (A) SMR Effect Plot (eQTL); (B) SMR Effect Plot (mQTL at cg08823240); (C) SMR Locus Plot (eQTL); (D) SMR Locus Plot (mQTL at cg08823240). [file Image3.tif]

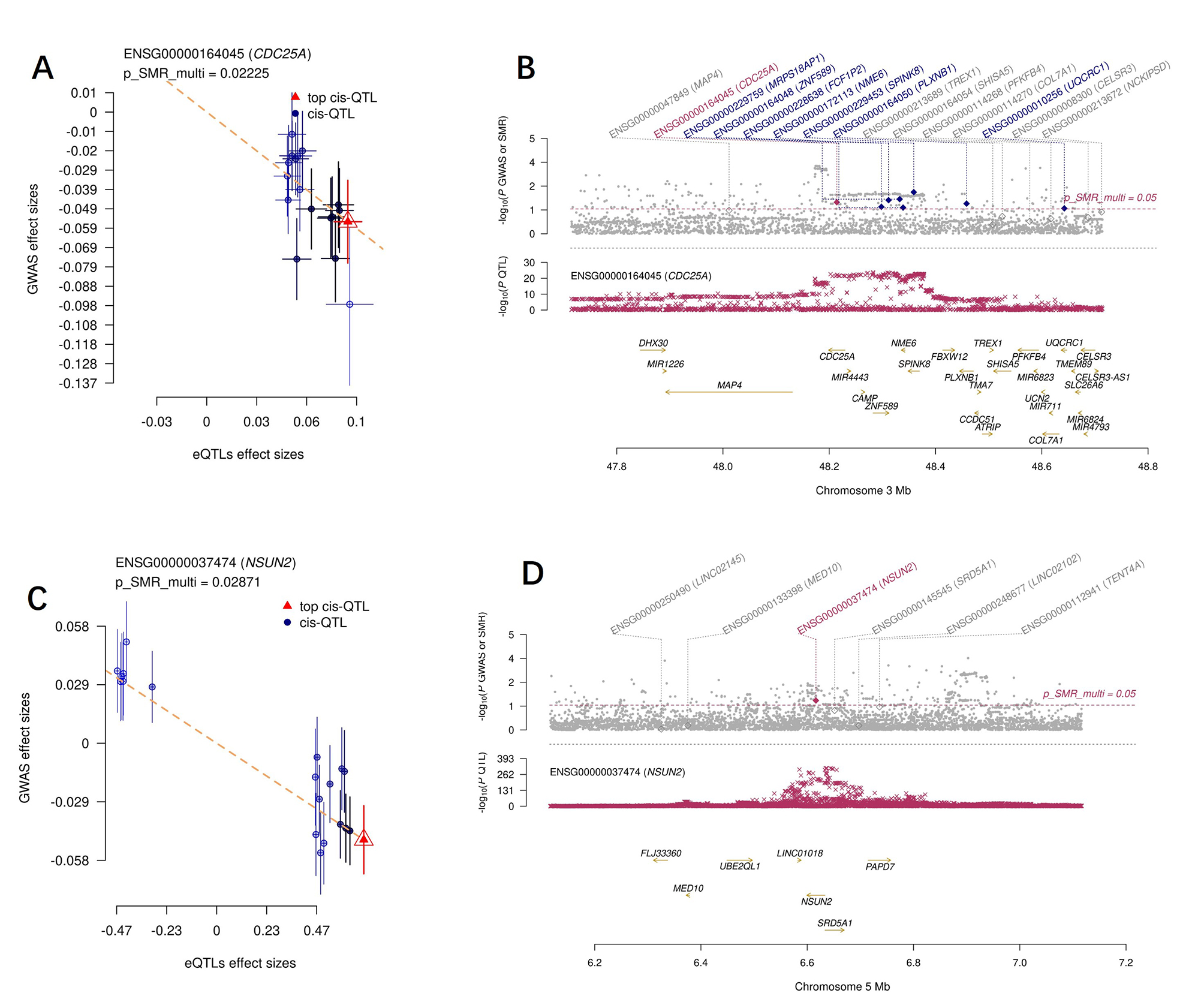

Supplement: Supplementary Figure 4 — SMRLocusPlot and SMREffectPlot for CDC25A, and NSUN2. (A) SMR Effect Plot of CDC25A in eQTL; (B) The SMR locus plot for CDC25A in eQTL; (C) SMR Effect Plot for NSUN2 in eQTL; (D) The SMR locus plot for NSUN2 at cg08823240 in eQTL. [file Image4.jpeg]
